# Costs of Care of HIV-Infected Children Initiating Lopinavir/Ritonavir-Based Antiretroviral Therapy before the Age of Two in Cote d’Ivoire

**DOI:** 10.1371/journal.pone.0166466

**Published:** 2016-12-09

**Authors:** Sophie Desmonde, Divine Avit, Junie Petit, Madeleine Amorissani Folquet, Francois Tanoh Eboua, Clarisse Amani Bosse, Evelyne Dainguy, Véronique Mea, Marguerite Timite-Konan, Sylvie Ngbeché, Andrea Ciaranello, Valeriane Leroy

**Affiliations:** 1 Inserm, U1219, Bordeaux University, Bordeaux, France; 2 Institut de Sante Publique, d’Epidemiologie et de Developpement, Bordeaux University, Bordeaux, France; 3 Medical Practice Evaluation Center, Department of Medicine, Massachusetts General Hospital, Boston, Massachusetts, United States of America; 4 Programme PACCI, Site ANRS, Abidjan, Cote d’Ivoire; 5 Service de Pediatrie, Centre Hospitalier Universitaire (CHU) de Cocody, Abidjan, Cote d’Ivoire; 6 Service de Pediatrie, Centre Hospitalier Universitaire (CHU) de Yopougon, Abidjan, Cote d’Ivoire; 7 Centre de Prise en charge, de recherche et de Formation (CePReF), Service Enfant, Yopougon, Abidjan, Cote d’Ivoire; 8 Division of Infectious Diseases, Massachusetts General Hospital, Boston, Massachusetts, United States of America; 9 Inserm Unit 1027, University of Toulouse 3, Toulouse, France; Universita degli Studi di Roma Tor Vergata, ITALY

## Abstract

**Objectives:**

To access the costs of care for Ivoirian children before and after initiating LPV/r-based antiretroviral therapy (ART) before the age of two.

**Methods:**

We assessed the direct costs of care for all HIV-infected children over the first 12 months on LPV/r-based ART initiated <2 years of age in Abidjan. We recorded all drug prescriptions, ART and cotrimoxazole prophylaxis delivery, medical analyses/examinations and hospital admissions. We compared these costs to those accrued in the month prior to ART initiation. Costs and 95% confidence intervals (95%CI) were estimated per child-month, according to severe morbidity.

**Results:**

Of the 114 children screened, 99 initiated LPV/r-based ART at a median age of 13.5 months (IQR: 6.8–18.6); 45% had reached World Health Organization stage 3 or 4. During the first 12 months on ART, 5% died and 3% were lost to follow-up. In the month before ART initiation, the mean cost of care per child-month reached $123.39 (95%CI:$121.02-$125.74). After ART initiation, it was $42.53 (95%CI:$42.15-$42.91); 50% were ART costs. The remaining costs were non-antiretroviral drugs (18%) and medical analyses/examinations (14%). Mean costs were significantly higher within the first three months on ART ($48.76, 95%CI:$47.95–$49.56) and in children experiencing severe morbidity ($49.76, 95%CI:$48.61–50.90).

**Conclusion:**

ART reduces the overall monthly cost of care of HIV-infected children < 2 years. Because children were treated at an advanced HIV disease stage, the additional costs of treating severe morbidity on ART remain substantial. Strategies for treating HIV-infected children as early as possible must remain a priority in Côte d’Ivoire.

## Introduction

Despite the progress in prevention of mother-to-child-transmission (PMTCT) programs, children continue to be infected with HIV. In 2013, 3.2 million children under the age of 15 were living with HIV globally, 91% of whom were in sub-Saharan Africa [1]. Côte d’Ivoire was one of the 21 priority countries in the Global Plan for the Elimination of New Pediatric HIV infections [2]. However, the decline of new infections is one of the slowest, reaching only 25% between 2009 and 2012 [3]. MTCT risks among HIV-exposed infants is therefore high, and 70,000 Ivorian children live with HIV [1]. In the absence of antiretroviral therapy (ART), 50% of HIV-infected children will die before their second birthday [4]. Benefits of early ART initiated before the age of 3 months have been demonstrated in the Children with Early Antiretroviral Therapy trial in South Africa in 2008 (the CHER trial), and in 2010 the World Health Organization (WHO) recommended systematic early ART initiation in all children <2 years of age [5,6]. These recommendations were further simplified in 2013, to include all children <5 years [7]. Although the number of children receiving ART has increased in all priority countries, the coverage in Côte d’Ivoire remains low, reaching only 16% in 2013 [1]. Furthermore, the WHO recommends initiating lopinavir/ritonavir (LPV/r)-based first-line ART in all children aged < 3 years [7–9]. However, the use of LPV/r raises a certain amount of difficulties in the scale-up of ART in resource-limited settings such as Côte d’Ivoire due to its storage conditions (difficulties in respecting the cold chain), unpalatable syrup formulations, potential long-term toxicity, interaction with tuberculosis drugs, and its expense [10].

As HIV care programs in Côte d’Ivoire are scaling up, policy-makers are addressing competing demands with limited resources. To prioritize interventions on improving care of HIV-infected children, it is critical to understand both the outcomes and costs of pediatric HIV care interventions. Costs analyses have been previously conducted in resource-limited settings; however these analyses are either not focused on a young pediatric population, do not include history of severe morbidity or World Health Organization (WHO) clinical stage, or do not include the West-African setting, and current projections are therefore being made with incomplete data [11–13]. We hypothesized that severe morbidity due to immunodeficiency would be higher in the pre-ART period inducing a higher cost of HIV-related morbidity care, while despite age, morbidity in the post-ART period would be lower due to the effect of ART on the immune-restoration expected within the first 6 months on ART and reversing the severe morbidity spectrum.

Thus, there is a need for accurate data on costs of delivering pediatric ART in West-Africa. These data, estimating patient costs of care will provide inputs for analyses modeling costs and cost-effectiveness of competing programmatic approaches [14,15]. This study assessed data on all costs of care in HIV-infected children during the first 12-months on LPV/r-based ART initiated before the age of 2 years, between 2011–2014, in Abidjan, Côte d’Ivoire. Secondly we described these costs of care in the month prior to ART initiation, and compared the costs 1-month prior to ART initiation and costs within 12-month of ART initiation according to their drugs categories.

## Methods

### Setting and follow-up

The MONOD ANRS 12206 project was a phase 3 non-inferiority randomized clinical trial conducted in Abidjan, Côte d’Ivoire and Ouagadougou, Burkina Faso (ClinicalTrial.gov registry number: NCT01127204) to assess whether LPV/r-based ART could be simplified by use of an Efavirenz (EFV)-based ART. Children with confirmed HIV-1 infection (two positive HIV-DNA PCR) tests, aged <24 months, free of tuberculosis and ART-naïve except for PMTCT interventions, whose both parents gave their written consent, were included [16]. They were first recruited in a 12-month “therapeutic cohort” at the time of ART initiation with 2 NRTIs (abacavir (ABC) or zidovudine (ZDV) and lamivudine (3TC)) and LPV/r given twice daily, together with cotrimoxazole as opportunistic infection prophylaxis. Then, children with virological success at 12 months (two HIV-RNA viral load measurements <500 copies/mL confirmed at least 3 months apart) were randomized to either switch to once-daily ABC-3TC-EFV therapy or stay on the twice-daily LPV/r regimen (AZT or ABC-3TC-LPV/r).

In this sub-study, we focused on the children enrolled between 2012 and 2013 in the initial therapeutic cohort from the Abidjan sites only: the *Centre de Prise en Charge et de Formation* in the district of Yopougon and the Abobo-Avocatier urban health clinic. Both sites were in the public sector. After an initial clinical visit, the parental consent was obtained, then children had a laboratory assessment and were initiated on ART combined with cotrimoxazole prophylaxis and therapeutic education. They had monthly clinical visits. Laboratory monitoring was consistent with Côte d’Ivoire guidelines: CD4, complete blood count, transaminases, glucose and creatinine were measured after 1 and 3 months and then every 6 months [17]. Viral load (HIV-RNA) was measured every three months. Children had access to clinical care for any incident morbidity. All costs of HIV and morbidity event care were covered by the project.

### Data collection

Over the follow-up period, we collected all available data on utilization of outpatient visits, antiretrovirals, cotrimoxazole prophylaxis, other medications, medical analyses/examinations (additional laboratory tests, medical imaging, and specialist visits), and hospitalizations (including costs of drugs and medical analyses/examinations during the stay). Costs of non-antiretroviral drugs and medical analyses/examinations were estimated through the trial budget records: each site provided the receipts or invoices and we used the actual charge of each resource reported on the invoices. All resources contributing to the patient’s clinical care were included, even if the cost was borne by another entity. In a routine care context, ART, cotrimoxazole and routine blood analyses are free of charge, supported by the National AIDS program; X-rays, inpatient care and non-ART medications are partly subsidized and routine laboratory tests (blood smears, cultures, microscopy) are fully paid for by the patient family (S1 and S2 Tables).

We also prospectively documented all severe morbid events during the 1-month pre-inclusion period (off ART) and within the first 12 months on LPV/r-based ART. We defined a severe morbid event (SME) as any fatal or life-threatening event, any event requiring hospitalization, any event leading to severe and lasting handicap, any event requiring an intervention to prevent an evolution to one of the conditions previously described, any event estimated as potentially severe by the trial investigator, and all grade 4 adverse clinical and biological events according to the Division of AIDS (DAIDS) classification of adverse events in children [18]. These data have been presented elsewhere [19]. All SME were validated by a pediatric committee.

### Primary outcome: cost per patient

We estimated the mean cost per patient-month for the first year on early LPV/r-based ART and evaluated the breakdown of mean cost per child-month of follow-up for each type of resource used. We estimated costs per child-month of care and their 95% confidence intervals (CI) overall for the cohort, as well as stratified by time on ART (first 3, 6 and 12 months after ART initiation) and by history of SME. Children contributed to the “No SME” period until date of their first event, from which point they contributed to the “History of SME” period until the end of their 12-month follow-up period. We computed Poisson CIs for mean costs per patient-month.

To determine the mean cost for outpatient visits per child-month, we summed the number of outpatient visits in the first 12 months and divided by the total number of patient-months to generate an average number of monthly visits. We then multiplied by the unit cost of an outpatient visit for Côte d’Ivoire, estimated by the WHO-CHOICE database (Choosing Interventions that are Cost Effective) [20]. To determine the mean cost for inpatient visits per child-month, the unit cost per inpatient visit was the sum of the total number of inpatient days over a given period divided by the total charges of hospitalizations invoiced for that same period.

Costs of cotrimoxazole and ARV drugs were not recorded in trial accounting documents because these medications were provided by national programs and free of charge for children in Côte d’Ivoire. We calculated these drug costs by multiplying the number of prescriptions by the unit prices listed in the Clinton Health Access Initiative (CHAI) price lists, according to drug formulation and dose [21].

Costs of non-antiretroviral drugs and medical analyses/examinations were calculated from a health care perspective. We used the financial accounting documents provided by each trial site, summed the total expenses for each type of drug or exam, and then divided by the total number of patient-months to generate a cost per patient-month of care for each type of drug and medical analyses/examinations overall.

We also performed the same estimations for the pre-inclusion period, between the first clinical contact and ART initiation. Data were collected as described above.

All costs were converted to 2012 US dollars at the rate of 589.20 Ivorian franc (XAF)/$1.

### Ethics

This study is within of the ANRS 12206 MONOD trial (ClinicalTrial.gov registry n°NCT01127204) which was approved by the Ethics committee for health research and the Health Ministry of Cote d’Ivoire (n°3323/MSLS/CNER-P).

## Results

### Inclusion process and pre-ART children characteristics

**Fig 1:**
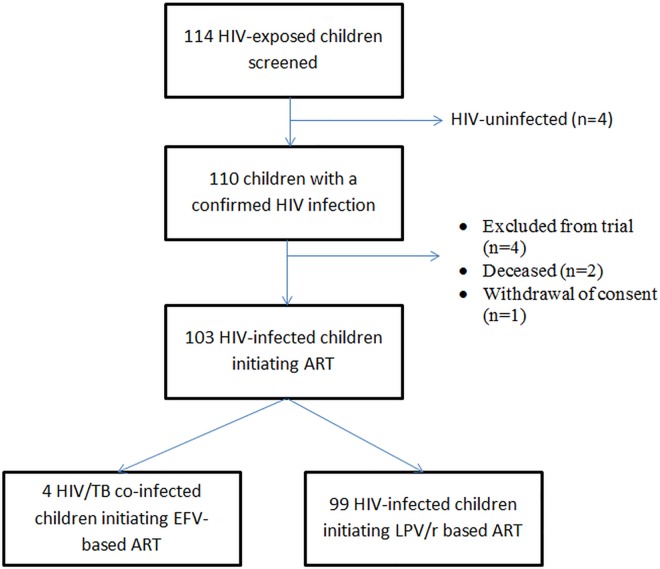
Flow diagram of the selection of the population.

From September 2011 to January 2013, 114 children were screened in participating sites for enrolment in the initial Monod cohort in Abidjan. The median age of these children at referral was 12 months (interquartile range (IQR): 7–18 months), 48% were female, and for 90% their mother was their primary caregiver. Of these children, 11 (9.6%) children were not included: in four, HIV was not confirmed on repeat testing (3.5%); two (1.8%) died before ART initiation, two (1.8%) had a severe anemia (Hb<7g/dL); one had an elevated transminases; one (0.8%) had a renal failure and one (0.8%) lacked parental consent. Among the 103 remaining children, four (10%) had tuberculosis co-infection and initiated a non-LPV/r-based regimen to permit tuberculosis treatment, and were not included in this cohort. Overall, we included 99 HIV-infected children initiating early LPV/r-based ART in this sub-study (Fig 1). In the pre-ART period, between screening and inclusion, SME rate was high among these children, reaching 21.37/100 child-months (95%CI: 13.63–33.50). Of these SME, two led to death; the mortality incidence rate was 2.37/100 child-months (95%CI: 2.04–2.67).

### Baseline characteristics and follow-up on ART

Among the 99 children included in the initial therapeutic cohort, 50 were girls; median time from screening to inclusion was 21 days (IQR: 14–28), and median age at inclusion was 13 months (IQR: 7–18). Of these children, 41% had already reached WHO Stage 3 or 4. The median percentage of CD4 cells was 20% [IQR: 15–27]. During the first 12 months on LPV/r-based ART, six (6%) children died and three (3%) were lost to follow-up. The mortality incidence rate on ART was 0.53 per 100 child-months (95%CI: 0.50–0.65). Overall, 24 SME were recorded in 22 children, with a SME rate reaching 2.12/ 100 child-months (95%CI: 2.04–2.21).

### Costs of care on ART

The total mean annual cost of care in these children on LPV/r-based ART during the first year is presented in Table 1: it was estimated at $552.68 (95%CI: $500–$605) per child. The mean cost per child-month of follow-up was $42.53 (95%CI: $42.15-$42.91).

**Table 1:** Mean costs of care estimated in Abidjan during the first 12-month on LPV/r based ART and per child-month of follow-up. N = 99. The MONOD ANRS 12206 study.

	12-month cost(2012 USD)		Cost per child-month(2012 USD)	
	Mean	95%CI*	Mean	*95%CI*
Outpatient visits	48.68	*[45*.*83–51*.*53]*	4.27	*[4*.*15–4*.*39]*
Inpatients visits	14.32	*[7*.*00–21*.*65]*	1.26	*[1*.*19–1*.*32]*
Drugs	87.65	*[76*.*53–98*.*77]*	7.69	*[7*.*53–7*.*85]*
Medical analyses/examinations	67.78	*[49*.*21–86*.*36]*	5.95	*[5*.*81–6*.*09]*
Cotrimoxazole	6.91	*[5*.*94–7*.*87]*	0.61	*[0*.*56–0*.*65]*
Antiretroviral drugs	259.52	*[244*.*26–274*.*79]*	22.76	*[22*.*49–23*.*04]*
Total	552.68	*[500*.*13–605*.*23]*	42.53	*[42*.*15–42*.*91]*

*CI: confidence interval

**Fig 2:**
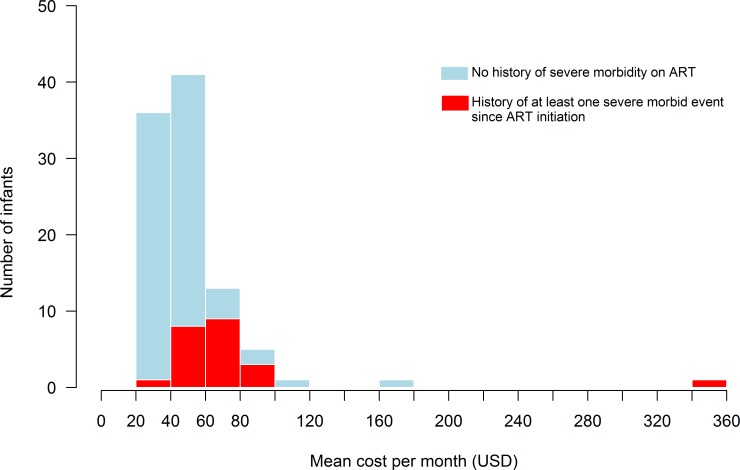
Distribution of the 99 children included in the first phase of the MONOD trial in Abidjan, Cote d’Ivoire, according to their mean cost per month of care during the first 12 months of LPV/r-based antiretroviral therapy, stratified by severe morbidity during follow-up (SME).

Most children had a monthly cost ranging from $20 to $100. Three children had a mean cost > $100 monthly; one of these children died 2 days after inclusion, following meningitis. His overall cost for the 2-day period of care was $58, which reported as a mean cost over 30 days, yields to $356. The two other children with mean monthly costs > $100 were both followed-up for the entire 12-month period and presented no SME; both had high medical analyses/examinations costs (>$600 over the year), mostly consultations with a specialist (Fig 2). The mean cost tended to be lower in the 77 children who had not experienced a SME compared to the 22 children who had, however this was not statistically significant ($518 [95%CI: $460 –$570] *vs* $670 [95%CI: $560 –$781], p = 0.8672).

ART represented the largest component of care costs (54%), followed by non-antiretroviral drugs (18%) and medical analyses/examinations (14%). Other drugs, medical analyses/examinations, and hospitalization costs were also substantial, reaching 32% of total costs in children who did not experience a SME and 54% in those who did experience SME. Table 2 reports the mean cost per child-month of follow-up according the occurrence of SME over the follow-up. Non-antiretroviral drugs and medical analyses/examinations were significantly more costly in children who had at least one SME ($8.99 per child-months, 95%CI:$8.49-$9.46 and $7.80 per child-months, 95%CI:$7.34-$8.24 respectively) compared to the no SME group ($7.48 per child-months, 95%CI:$7.31-$7.65 and $5.66 per child-months, 95%CI:$5.51 -$5.81 respectively). There was no significant difference in the cost of antifungal/parasitic medication or iron supplementation between both groups. However, we observed higher costs of antibiotics and other drugs in children who had at least one SME during follow-up. We also note that children with history of SME reported lower costs of cotrimoxazole, $0.43 per child-months (95%CI:$0.32-$0.52) and ART, $18.06 per child-months (95%CI:$17.36-$18.74) compared to $0.63 per child-months (95%CI:$0.58-$0.68) and $23.67per child-months (95%CI:$23.37-$23.97) in children with no history of SME, possibly indicating lower resource use among children after SME.

**Table 2:** Mean costs per child-month according the Severe Morbid Event (SME) history in HIV-infected children initiating LPV/r–based ART< 24 months of age in Abidjan, Cote d’Ivoire. The MONOD ANRS 12206 study.

	No history of SME*		At least one SME* during follow-up	
	Cost per child-month(2012 USD)	*95%CI*	Cost per child-month(2012 USD)	*95%CI*
Outpatient visits	4.21	*[4*.*08–4*.*34]*	4.60	*[4*.*25–4*.*94]*
Inpatients visits	0.00		9.89	*[9*.*37–10*.*39]*
Drugs	7.48	*[7*.*31–7*.*65]*	8.99	*[8*.*49–9*.*46]*
Antibiotics	1.48	*[1*.*41–1*.*56]*	2.02	*[1*.*79–2*.*24]*
Antifungals	0.77	*[0*.*72–0*.*82]*	0.93	*[0*.*78–1*.*08]*
Iron Supplements	1.76	*[1*.*68–1*.*84]*	1.47	*[1*.*27–1*.*66]*
Other	3.47	*[3*.*35–3*.*58]*	4.56	*[4*.*21–4*.*89]*
Medical analyses/examinations	5.66	*[5*.*51–5*.*81]*	7.80	*[7*.*34–8*.*24]*
Cotrimoxazole	0.63	*[0*.*58–0*.*68]*	0.43	*[0*.*32–0*.*52]*
Antiretroviral drugs	23.67	*[23*.*37–23*.*97]*	18.06	*[17*.*36–18*.*74]*
Total	41.65	*[41*.*25–42*.*06]*	49.76	*[48*.*61–50*.*90]*

*SME: severe morbid event

The mean cost per child-month according to time since ART initiation is described in Table 3. This was substantially higher during the first 3 months of ART than in later periods, reaching a mean cost of $48.76 per child-months (95%CI: $47.95–$49.56) compared to $38.84 per child-months (95%CI: $38.11–$39.56) at 3–6 months post-ART initiation and $41.19 per child-months (95%CI: $40.65–$41.72) at 6–12 months post ART initiation. Furthermore, during the first 3 months on ART, non-ART costs were higher than during later periods: inpatient care costs ($2.51 per child-months (95%CI: $2.33-$2.69) vs $0.98 per child-months (95%CI: $0.90-$1.06) and non-ARV/non-CTX drugs ($11.21 per child-months (95%CI: $10.83-$11.59) compared to $6.63 per child-months (95%CI: $6.42-$6.85) were > 2-fold higher during the first 3 months of ART compared to 6–12 months after ART initiation (Table 3).

**Table 3:** Mean 12-month cost and mean costs per child-months at 0–3, 3–6 and 6–12 months since LPV/r–based ART initiation in children < 24 months of age in Abidjan, Cote d’Ivoire. The MONOD ANRS 12206 study.

	0–3 months after ART initiation		3–6 months after ART initiation		6–12 months after ART initiation	
	Cost per child-month	*95%CI*	Cost per child-month	*95%CI*	Cost per child-month	*95%CI*
Outpatient visits	4.84	*[4*.*59–5*.*09]*	4.05	*[3*.*82–4*.*28]*	4.09	*[3*.*92–4*.*25]*
Inpatients visits	2.51	*[2*.*33–2*.*69]*	0.52	*[0*.*43–0*.*60]*	0.98	*[0*.*90–1*.*06]*
Drugs	11.21	*[10*.*83–11*.*59]*	6.17	*[5*.*88–6*.*45]*	6.63	*[6*.*42–6*.*85]*
Medical analyses/examinations	6.82	*[6*.*52–7*.*12]*	4.73	*[4*.*48–4*.*98]*	6.11	*[5*.*91–6*.*32]*
Cotrimoxazole	0.61	*[0*.*52–0*.*69]*	0.61	*[0*.*52–0*.*69]*	0.61	*[0*.*54–0*.*67]*
Antiretroviral drugs	22.76	*[22*.*21–23*.*31]*	22.76	*[22*.*21–23*.*31]*	22.76	*[22*.*37–23*.*16]*
Total	48.76	*[47*.*95–49*.*56]*	38.84	*[38*.*11–39*.*57]*	41.19	*[40*.*65–41*.*72]*

### Comparison of costs of care before and after ART initiation

Table 4 presents the costs of care per child in the month prior to ART initiation and stratified by history of SME. The overall mean cost in that month was $123.39 per child (95%CI: 121.02–125.74). Despite the additional costs of ART, the overall monthly costs of care are substantially lower in children after ART initiation compared to the month preceding ART initiation. The costs of ART, once initiated, are offset by the use of medical analyses/examinations, drugs, and hospitalization. In children with history of SME, the monthly cost of hospitalizations was $ 149.80 (95%CI: $ 141.75 –$ 157.63 before ART initiation and $9.89 (95%: $9.37–$10.39) after ART initiation (Table 2 and Table 4).

**Table 4:** Mean costs per child-month overall and according the Severe Morbid Event (SME) history in HIV-infected children within 1 month prior to ART in Abidjan, Cote d’Ivoire. The MONOD ANRS 12206 study.

	Overall		No history of SME		At least one SME during follow-up	
	Cost per child-month	*95%CI*	Cost per child-month	*95%CI*	Cost per child-month	*95%CI*
Outpatient visits	12.05	*[11*.*31–12*.*77]*	11.90	*[11*.*12–12*.*65]*	13.34	*[10*.*93–15*.*51]*
Inpatients visits	15.75	*[14*.*90–16*.*57]*	0		149.80	*[141*.*96–157*.*63]*
Drugs	60.53	*[58*.*87–62*.*16]*	65.67	*[63*.*84–67*.*47]*	16.77	*[14*.*07–19*.*23]*
Medical analyses/examinations	35.06	*[33*.*80–36*.*30]*	36.64	*[35*.*27–37*.*98]*	21.66	*[18*.*60–24505]*
Total	123.39	*[121*.*02–125*.*74]*	114.21	*[111*.*80–116*.*59]*	201.56	*[192*.*23–210*.*68]*

## Discussion

This study is one of the first to document current costs of care in a pediatric population initiated on LPV/r-based ART before two years of age in West Africa, with several key findings. First, we estimated the overall cost of care to be $552 per child or $42.53 per child-month over the first year on ART. Approximately 50% of these costs were due to antiretroviral drugs, with the remainder attributable to other drugs (18%), medical analyses/examinations (14%) and outpatient care (10%). Second, we found that costs of hospitalizations and non-antiretroviral drugs were 2-fold higher within the first 3 months of ART initiation compared to later periods. Third, although there was no significant difference in overall costs in children with and without history of SME over the first 12 months on ART, the monthly costs of non-ART drugs (specifically antibiotics) and medical analyses/examinations were higher in children with a history of SME. Finally, when comparing costs before and after ART initiation, we observed a significant 3-fold decrease in costs of care per child-month after ART initiation, despite the additional costs of antiretroviral drugs.

The estimated mean overall 12-month cost of care of $552 was lower than has been reported for ART programs in other settings. In the CHER trial in South Africa, the cost of pediatric care was estimated at $770 during the first year on ART (12). We explain these differences mainly by the higher GDP *per capita* in South Africa (estimated at $6,500 in 2014) compared to Cote d’Ivoire (estimated at $1,500 in 2014) and the differences in unit cost estimates in the WHO-CHOICE database [20,22] which are higher in the South African setting. Our results are, however, comparable to those of the ARROW trial, investigating alternative strategies to deliver pediatric ART in Zimbabwe and Uganda, two similar settings to Côte d’Ivoire from an economic point of view. The ARROW trial reported an overall mean cost ranging from $1775–$2328 for a 4-year period, thus a mean of $443–$581 for 12 months on ART [23].

We observed that less than half of the costs of care in HIV-infected children were antiretroviral drug costs, reaching $22 per child-month in our study. This is comparable to those reported for Côte d’Ivoire in the WHO global price reporting mechanism database [24]. This suggests that children are still experiencing a substantial amount of residual non-severe morbidity while on ART, contributing to the remaining 50% of monthly costs of care. Indeed non-antiretroviral drugs represented 18% of overall costs per patient-month. Iron supplements and other vitamins were the majority of the prescriptions, suggesting that children were also experiencing nutritional deficiencies prior to ART initiation. Previous studies have reported high rates of the prevalence of malnutrition in HIV-infected children at ART initiation, particularly in West-Africa [25–27].

As expected, we observed substantially higher monthly costs of care in children with a history of SME compared to those without. The proportion of non-ART costs of overall costs was lower in the SME group, compared to the non-SME group (36% vs. 57%),. Among children with SME history, 20% of costs were due to hospitalizations. Based on reported charges in the Monod trial, the mean cost of hospitalization was $9.78 per inpatient-day. This is higher than the WHO-CHOICE estimate for Cote d’Ivoire for a tertiary hospital, but lower than the $34 reported in the ARROW trial setting [20,23]. We also noted that children with a history of SME during follow-up had a significantly lower cost of cotrimoxazole prophylaxis and ART per patient-month. This suggests that the sickest children had used less prophylaxis and ART, but utilized higher rates of hospitalizations and non-antiretroviral drugs. Since the ART costs were derived according to the exact delivery to the patients in the trial, we hypothesize that these children may have been less adherent to their ART treatment and follow-up.

We reported significantly higher non-ART costs within the first three months of ART initiation, compared to later periods. This was mainly due to the high morbidity and mortality in these children who were largely symptomatic at ART initiation, reflecting late access to ART in Côte d’Ivoire as reported elsewhere [28]. For example, non-ART medication costs were 3-fold higher during this period, and costs of antibiotics and antifungals were 2-fold higher within those first three months on ART. We explain this by the high morbidity rate among HIV-infected children within the first months of ART initiation [29,30]. The overall non-ART cost of care averaged $20 per child-month. Although these costs were paid by the trial, in a routine care context these costs would be borne by the patients’ families. $20/month represents 20% of the average salary in Cote d’Ivoire which was estimated to be $102 / month in 2012 [31]. In a country where the annual health expenditure per habitant is estimated to be $88, healthcare costs borne by families of HIV-infected children remain substantial.

When comparing the mean costs per child-month to those estimated in the month prior to ART initiation, we observe a significant drop in costs after ART initiation. Despite the expense of ART, the overall costs per child-month decreased by approximately 60% after treatment initiation, regardless of history of SME. The CHER trial showed a similar short-term reduction in overall costs, due to reduction of severe morbidity and thus healthcare resource utilization and costs [5]. When considered over longer time horizons, ART does not save money, because children live longer and require years of medications and care, but the long term treatment of HIV-infected children and adults has been shown to be very “cost-effective,” or an excellent value for healthcare spending [14,32–34].

In our study, 45% of children initiating ART were already presenting with WHO stage 3 or 4 conditions suggesting that these children were initiating ART at a clinically advanced stage of disease and thus experiencing severe morbidity, as shown in previous studies [28,29,35–37]. Younger children are particularly vulnerable to severe morbidity despite ART, underscoring the urgent need for early identification, and rapid cotrimoxazole and ART initiation. This will not only improve clinical outcomes but also reduce the costs of care, many of which are borne by patient families in the field conditions.

Our study has a number of limitations. First, the study was conducted within a research context, where the resource utilization may be higher than in routine care. Nevertheless, previous studies conducted in routine care programs in Cote d’Ivoire have reported high rates of healthcare resource utilization suggesting that HIV-infected children do require substantial utilization of healthcare service despite their costs [29,38]. Furthermore, we acknowledge that there was no control group of untreated HIV-infected children to directly compare costs, not confounded by age. It would have been unethical to follow simultaneously a group of known HIV-infected children of the same age and not initiating them on ART. Therefore, our design was observational, assessing mean monthly costs within 12 months of ART initiation as well as in the month prior to ART initiation. The pre- ART period was limited to a “one-month” evaluation period only (as opposed to the 12-month post ART period) as it represented the time between HIV diagnosis and ART initiation. We were unable to perform direct pre and post ART comparisons because of the temporal variations and this 1-month period is not representative of the whole pre-ART period. Indeed, because of the low EID coverage in this setting, the children included in the MONOD cohort were for the most part identified as HIV-infected following severe morbidity. They most likely remained asymptomatic until then and extrapolating the rates we observe in this 1-month to the whole pre-ART period would be biased. Furthermore many children died prior to diagnosis while others are still asymptomatic and unidentified as HIV-infected. Second, estimations are based on a relatively small sample of 99 children who initiated LPV/r, which is a drug still difficult to offer, and mainly reserved for early treatment in children less than three and for second-line regimens in Cote d’Ivoire. Our data may not be representative of the costs and outcomes expected in routine care, where many children still receive a Nevirapine-based triple therapy. However, since 2013, Côte d’Ivoire recommends routine treatment based on LPV for all children less than three years old. Cost comparison of LPV versus EFV or NNRTI-based ART regimens will be further analyzed. Third, results represent a mean cost per child-month over a short period, and only direct costs were considered. They do not reflect the overall costs for the implementation of early LPV/r-based ART, nor the long-term costs related to possible toxicity or poor adherence. However, our results are consistent with findings from other pediatric HIV treatment programs, again supporting the urgent need to increase access to ART the earliest possible [15,39–42].

Our study provides policy-makers in Cote d’Ivoire with original and relevant estimates of the costs related to the first year of pediatric LPV/r-based ART. This is of importance for future national guidelines as LPV/r becomes more widely accessible as a first-line drug in the management of pediatric HIV in West Africa. The high resource use and associated costs in the month prior to ART initiation suggest that these children were already very ill at time of inclusion. Because children were treated at an advanced HIV-disease stage, the additional costs of treating severe morbidity on ART remain substantial. These results are generalizable to other settings in Western and Central Africa, with low to moderate HIV prevalence but where EID uptake remains poor. Implementing the WHO guidelines for early infant diagnosis and ART initiation with a focus on the youngest children still remains a priority in Cote d’Ivoire.

## Supporting Information

S1 Table Breakdown of the unit costs of medication in Abidjan, Côte d’Ivoire (2012). (DOCX)

S2 Table Breakdown of the unit costs of complementary exams in Abidjan, Côte d’Ivoire (2012) (DOCX)
