# Supplementary material for: Costs of Care of HIV-Infected Children Initiating Lopinavir/Ritonavir-Based Antiretroviral Therapy before the Age of Two in Cote d’Ivoire
Source: PLoS One. 2016 Dec 9;11(12):e0166466. doi: 10.1371/journal.pone.0166466 (PMC5147813; doi:10.1371/journal.pone.0166466)
Supplement: S1 Table — (DOCX) [file pone.0166466.s001.docx]

Supporting information

Table 1 – Breakdown of the unit costs of medication in Abidjan, Côte d’Ivoire (2012)

| **Drug name (French denomination)** | **Treatment class** | **Cost in Ivorian francs (2012)** | **Cost in US DOLLARS (2012)** |
| --- | --- | --- | --- |
| *ASPEGIC* | Analgesic | 1000 | 1,70 |
| *ASPIRINE* | Analgesic | 820 | 1,39 |
| *BRUSTAN* | Analgesic | 1260 | 2,14 |
| *BUFENOL* | Analgesic | 1105 | 1,88 |
| *DOLIPRANE* | Analgesic | 1095 | 1,86 |
| *DOLKO* | Analgesic | 1180 | 2,00 |
| *EFFADOL* | Analgesic | 1095 | 1,86 |
| *EFFERALGAN* | Analgesic | 1245 | 2,11 |
| *NOVALGIN* | Analgesic | 1000 | 1,70 |
| *PANADOL* | Analgesic | 1050 | 1,78 |
| *PANOL* | Analgesic | 730 | 1,24 |
| *PANSORAL PREMIERES DENTS* | Analgesic | 2880 | 4,89 |
| *PARACETAMOL* | Analgesic | 1260 | 2,14 |
| *BACTROBAN* | Anti-infective | 4830 | 8,20 |
| *BANEOCIN* | Anti-infective | 1565 | 2,66 |
| *AERIUS* | Allergy treatments | 3965 | 6,73 |
| *COFANTRINE* | Allergy treatments | 1050 | 1,78 |
| *NAAXIA* | Allergy treatments | 3200 | 5,43 |
| *PAIDOTERIN* | Allergy treatments | 2530 | 4,29 |
| *PRIMALAN* | Allergy treatments | 2015 | 3,42 |
| *ZYRTEC* | Allergy treatments | 6395 | 10,85 |
| *ACLAV* | Antibiotics | 3235 | 5,49 |
| *AMITRON* | Antibiotics | 1200 | 2,04 |
| *AMODEX* | Antibiotics | 1280 | 2,17 |
| *AMOXICILLINE* | Antibiotics | 905 | 1,54 |
| *ASTAPH* | Antibiotics | 3205 | 5,44 |
| *AUGMENTIN* | Antibiotics | 4595 | 7,80 |
| *AURICULARUM* | Antibiotics | 6800 | 11,54 |
| *BACTOX* | Antibiotics | 1670 | 2,83 |
| *BACTRIM* | Antibiotics | 2085 | 3,54 |
| *BIODROXIL* | Antibiotics | 3115 | 5,29 |
| *CAMOQUIN PLUS* | Antibiotics | 3000 | 5,09 |
| *CEBEMYXINE* | Antibiotics | 2230 | 3,78 |
| *CEDROX* | Antibiotics | 2985 | 5,07 |
| *CEFTRIAXONE* | Antibiotics | 650 | 1,10 |
| *CEPHACET* | Antibiotics | 1000 | 1,70 |
| *CETRAXAL* | Antibiotics | 4620 | 7,84 |
| *CETRAXAL OTICO* | Antibiotics | 4510 | 7,65 |
| *CIPHACET* | Antibiotics | 1000 | 1,70 |
| *CLAMOXYL* | Antibiotics | 1130 | 1,92 |
| **Drug name (French denomination)** | **Treatment class** | **Cost in Ivorian francs (2012)** | **Cost in US DOLLARS (2012)** |
| *CO-AMOXI NN* | Antibiotics | 3640 | 6,18 |
| *COARTEM* | Antibiotics | 1310 | 2,22 |
| *COTREX* | Antibiotics | 830 | 1,41 |
| *CURAM* | Antibiotics | 5435 | 9,22 |
| *DUPLAMOX* | Antibiotics | 3630 | 6,16 |
| *ERY* | Antibiotics | 2850 | 4,84 |
| *FLOXAPEN* | Antibiotics | 7200 | 12,22 |
| *FRAKIDEX* | Antibiotics | 1565 | 2,66 |
| *FUCIDINE* | Antibiotics | 2285 | 3,88 |
| *FUCITHALMIC* | Antibiotics | 2705 | 4,59 |
| *GENTA* | Antibiotics | 1340 | 2,27 |
| *GENTALLINE* | Antibiotics | 990 | 1,68 |
| *HICONCIL* | Antibiotics | 1485 | 2,52 |
| *JOSACINE* | Antibiotics | 5160 | 8,76 |
| *MEGACEF* | Antibiotics | 3400 | 5,77 |
| *OFLOCET* | Antibiotics | 5435 | 9,22 |
| *OFRAMAX* | Antibiotics | 4030 | 6,84 |
| *ORACEFAL* | Antibiotics | 3165 | 5,37 |
| *OROKEN* | Antibiotics | 9345 | 15,86 |
| *OTAFA* | Antibiotics | 2810 | 4,77 |
| *POLYDEXA* | Antibiotics | 1800 | 3,05 |
| *RIFAMYCINE* | Antibiotics | 2495 | 4,23 |
| *ROCEPHINE* | Antibiotics | 650 | 1,10 |
| *TOBREX* | Antibiotics | 2250 | 3,82 |
| *ZAMOX* | Antibiotics | 4590 | 7,79 |
| *ACTAPULGITE* | Antidiarrheal treatments | 1750 | 2,97 |
| *APASIDE* | Antidiarrheal treatments | 1790 | 3,04 |
| *ERCEFURYL* | Antidiarrheal treatments | 3220 | 5,47 |
| *LACTEOL FORT* | Antidiarrheal treatments | 3060 | 5,19 |
| *OSMULE* | Antidiarrheal treatments | 280 | 0,48 |
| *CANCID* | Antifungal agents | 1895 | 3,22 |
| *DAKTARIN* | Antifungal agents | 4545 | 7,71 |
| *ECOREX* | Antifungal agents | 2665 | 4,52 |
| *EXOFENE POUDRE* | Antifungal agents | 2440 | 4,14 |
| *FLUCAZOL* | Antifungal agents | 2230 | 3,78 |
| *FLUCONAZOLE* | Antifungal agents | 2230 | 3,78 |
| *FUNGIZONE* | Antifungal agents | 6220 | 10,56 |
| *GRISEO* | Antifungal agents | 1030 | 1,75 |
| *KETODERM* | Antifungal agents | 3940 | 6,69 |
| *MICOZAL* | Antifungal agents | 1680 | 2,85 |
| *MICOZOLE* | Antifungal agents | 1600 | 2,72 |
| *PEVARYL* | Antifungal agents | 3460 | 5,87 |
| *ADVIL* | Anti-inflammatory treatments | 2525 | 4,29 |
| *BETASONE* | Anti-inflammatory treatments | 3165 | 5,37 |
| *BRUFEN* | Anti-inflammatory treatments | 3200 | 5,43 |
| **Drug name (French denomination)** | **Treatment class** | **Cost in Ivorian francs (2012)** | **Cost in US DOLLARS (2012)** |
| *CELESTENE* | Anti-inflammatory treatments | 4000 | 6,79 |
| *IBUPAR* | Anti-inflammatory treatments | 970 | 1,65 |
| *NIFLURIL* | Anti-inflammatory treatments | 1395 | 2,37 |
| *SOLUPRED* | Anti-inflammatory treatments | 2445 | 4,15 |
| *ARTEFON* | Anti-malarial drugs | 2655 | 4,51 |
| *ARTEMETHER* | Anti-malarial drugs | 410 | 0,70 |
| *ARTEPAL* | Anti-malarial drugs | 2670 | 4,53 |
| *ARTESIANE* | Anti-malarial drugs | 3000 | 5,09 |
| *COARTESIANE* | Anti-malarial drugs | 3800 | 6,45 |
| *DARTE-Q* | Anti-malarial drugs | 750 | 1,27 |
| *FANTEM* | Anti-malarial drugs | 1500 | 2,55 |
| *LARITEM* | Anti-malarial drugs | 735 | 1,25 |
| *LUFANTER* | Anti-malarial drugs | 1520 | 2,58 |
| *LUMARTEM* | Anti-malarial drugs | 2640 | 4,48 |
| *MALACURE* | Anti-malarial drugs | 3965 | 6,73 |
| *PLASMOCID* | Anti-malarial drugs | 1310 | 2,22 |
| *A-PAR* | Antiparasitics | 4180 | 7,09 |
| *ACARIBIAL* | Antiparasitics | 2600 | 4,41 |
| *ALBEN* | Antiparasitics | 1540 | 2,61 |
| *FLAGYL* | Antiparasitics | 2510 | 4,26 |
| *FLUVERMAL* | Antiparasitics | 2800 | 4,75 |
| *HELMINTHOX (PYRANTEL)* | Antiparasitics | 1350 | 2,29 |
| *METHODEX* | Antiparasitics | 2110 | 3,58 |
| *METRONIDAZOLE* | Antiparasitics | 765 | 1,30 |
| *SPREGAL* | Antiparasitics | 4220 | 7,16 |
| *TOLOXIN* | Antiparasitics | 1530 | 2,60 |
| *VEREX* | Antiparasitics | 1000 | 1,70 |
| *VERMOX* | Antiparasitics | 1895 | 3,22 |
| *VERZOL* | Antiparasitics | 940 | 1,60 |
| *ACIDE BORIQUE* | Antiseptics | 2300 | 3,90 |
| *BACTYL* | Antiseptics | 1140 | 1,93 |
| *BETADINE* | Antiseptics | 1205 | 2,05 |
| *BETADINE JAUNE* | Antiseptics | 1495 | 2,54 |
| *BETADINE VERTE* | Antiseptics | 1575 | 2,67 |
| *COLLUNOVAR* | Antiseptics | 2000 | 3,39 |
| *CYTEAL* | Antiseptics | 2300 | 3,90 |
| *DACRYOSERUM* | Antiseptics | 3015 | 5,12 |
| *DAKIN COOPER* | Antiseptics | 1445 | 2,45 |
| *DERMOBACTER* | Antiseptics | 1800 | 3,05 |
| *DESOMEDINE* | Antiseptics | 1800 | 3,05 |
| *DEXAMETHASONE* | Antiseptics | 500 | 0,85 |
| *EOSINE* | Antiseptics | 1620 | 2,75 |
| *MERCUROCHROME* | Antiseptics | 1500 | 2,55 |
| *PRORHINEL* | Antiseptics | 1380 | 2,34 |
| *PYRALVEX* | Antiseptics | 2730 | 4,63 |
| **Drug name (French denomination)** | **Treatment class** | **Cost in Ivorian francs (2012)** | **Cost in US DOLLARS (2012)** |
| *SEPTILAIT* | Antiseptics | 2089 | 3,55 |
| *SILVEREX* | Antiseptics | 8245 | 13,99 |
| *ATARAX* | Anxiolytics | 1905 | 3,23 |
| *TRESORIX* | Anxiolytics | 2275 | 3,86 |
| *AEROSOL* | Other | 4180 | 7,09 |
| *APDYL-H* | Other | 1520 | 2,58 |
| *APTEYL* | Other | 2640 | 4,48 |
| *ARGININE SIROP* | Other | 4525 | 7,68 |
| *ARGININE VEYRON* | Other | 4523 | 7,68 |
| *AURIPAX* | Other | 1705 | 2,89 |
| *BABYSOIN* | Other | 1495 | 2,54 |
| *BIAFINE* | Other | 2030 | 3,45 |
| *BICARBONATE* | Other | 2040 | 3,46 |
| *BRONCALENE* | Other | 2480 | 4,21 |
| *BRONCHODERMINE* | Other | 1615 | 2,74 |
| *CANDIBIOTIC* | Other | 2535 | 4,30 |
| *CARBEN* | Other | 1285 | 2,18 |
| *CEPODEM* | Other | 6030 | 10,23 |
| *CERULYSE* | Other | 1970 | 3,34 |
| *COQUELUSEDAL* | Other | 1180 | 2,00 |
| *DEBRIDAT* | Other | 2245 | 3,81 |
| *DECODERM* | Other | 4295 | 7,29 |
| *DEXERYL* | Other | 3610 | 6,13 |
| *DIPROSONE* | Other | 3020 | 5,13 |
| *EDUCTYL* | Other | 1350 | 2,29 |
| *EXOMUC* | Other | 3465 | 5,88 |
| *FIXIM* | Other | 6995 | 11,87 |
| *FLUDITEC* | Other | 1585 | 2,69 |
| *GENTASOL COLLYRE* | Other | 1040 | 1,77 |
| *HOMEOPLASMINE* | Other | 4130 | 7,01 |
| *ISOFRA* | Other | 1535 | 2,61 |
| *LEVOPHYTA* | Other | 4950 | 8,40 |
| *LIDOCAINE* | Other | 1100 | 1,87 |
| *LITACOLD* | Other | 1070 | 1,82 |
| *LOCAPRED* | Other | 18855 | 32,00 |
| *MARIMER* | Other | 3935 | 6,68 |
| *MAXILASE* | Other | 2425 | 4,12 |
| *MOTILIUM* | Other | 2575 | 4,37 |
| *MUCOMYST* | Other | 2480 | 4,21 |
| *ORICEDAL* | Other | 1585 | 2,69 |
| *OTRIVINE* | Other | 1475 | 2,50 |
| *OXYPLASTINE* | Other | 1800 | 3,05 |
| *PANOTILE* | Other | 1735 | 2,94 |
| *PAROL* | Other | 1585 | 2,69 |
| *PASSEDYL* | Other | 2200 | 3,73 |
| **Drug name (French denomination)** | **Treatment class** | **Cost in Ivorian francs (2012)** | **Cost in US DOLLARS (2012)** |
| *PERIDYS* | Other | 2575 | 4,37 |
| *PHYSIODOSE* | Other | 2100 | 3,56 |
| *PIVALONE* | Other | 2865 | 4,86 |
| *PNEUMOREL* | Other | 1450 | 2,46 |
| *POSITON* | Other | 2890 | 4,90 |
| *PRUREX* | Other | 1455 | 2,47 |
| *RHINATHIOL* | Other | 1995 | 3,39 |
| *SAFORELLE* | Other | 3830 | 6,50 |
| *SERUM PHYSIOLOGIQUE* | Other | 2100 | 3,56 |
| *SMECTA* | Other | 1445 | 2,45 |
| *SPASFON LYOC* | Other | 2520 | 4,28 |
| *SPASMOBUTINE* | Other | 2480 | 4,21 |
| *STERIMAR* | Other | 2520 | 4,28 |
| *TANAGEL* | Other | 3015 | 5,12 |
| *TERPONE* | Other | 1830 | 3,11 |
| *TRIDESONIT* | Other | 1710 | 2,90 |
| *TRIMETABOL* | Other | 2965 | 5,03 |
| *ULTRA LEVURE* | Other | 3673 | 6,23 |
| *VENTOLINE* | Other | 3250 | 5,52 |
| *VIBROCIL* | Other | 1995 | 3,39 |
| *VOGALENE* | Other | 1750 | 2,97 |
| *XYLO-MEPHA* | Other | 1275 | 2,16 |
| *XYLOCAINE* | Other | 1200 | 2,04 |
| *ZOLEM* | Other | 1225 | 2,08 |
| *CATHETER* | Medical supplies | 700 | 1,19 |
| *COMPRESSES* | Medical supplies | 1130 | 1,92 |
| *GANTS STERILES* | Medical supplies | 750 | 1,27 |
| *INTRACANULE* | Medical supplies | 210 | 0,36 |
| *INTRANULE G24* | Medical supplies | 610 | 1,04 |
| *LAME DE BISTOURI* | Medical supplies | 300 | 0,51 |
| *LEUKOPLAST* | Medical supplies | 2665 | 4,52 |
| *MATERIEL DE TRANSFUSION* | Medical supplies | 505 | 0,86 |
| *MATERIEL DE TUBAGE* | Medical supplies | 10000 | 16,97 |
| *PERFUSEUR* | Medical supplies | 500 | 0,85 |
| *SERINGUE* | Medical supplies | 450 | 0,76 |
| *SONDE GASTRIQUE* | Medical supplies | 965 | 1,64 |
| *SONDE NASO-GASTRIQUE* | Medical supplies | 980 | 1,66 |
| *TRANSFUSEUR* | Medical supplies | 750 | 1,27 |
| *CELIA DEVELOP SRO* | Rehydration | 260 | 0,44 |
| *NICICALCIUM* | Rehydration | 2580 | 4,38 |
| *NaCl* | Rehydration | 425 | 0,72 |
| *ORASEL* | Rehydration | 160 | 0,27 |
| *RINGER LACTATE* | Rehydration | 950 | 1,61 |
| *S.R.O* | Rehydration | 160 | 0,27 |
| *SERUM GLUCOSE ISOTONIQUE* | Rehydration | 810 | 1,37 |
| **Drug name (French denomination)** | **Treatment class** | **Cost in Ivorian francs (2012)** | **Cost in US DOLLARS (2012)** |
| *GELOPLASMA* | Infusion solution | 3975 | 6,75 |
| *PERFALGAN* | Infusion solution | 2165 | 3,67 |
| *AMIFER* | Iron supplementation | 2755 | 4,68 |
| *BIO-FER* | Iron supplementation | 2885 | 4,90 |
| *FER UCB AB* | Iron supplementation | 4080 | 6,92 |
| *FERAMALT* | Iron supplementation | 6100 | 10,35 |
| *FERCEFOL* | Iron supplementation | 1575 | 2,67 |
| *FERROLEX* | Iron supplementation | 1575 | 2,67 |
| *FERROSTRANE* | Iron supplementation | 1830 | 3,11 |
| *FUMAFER* | Iron supplementation | 1595 | 2,71 |
| *NOVAFER* | Iron supplementation | 3180 | 5,40 |
| *RANFERON* | Iron supplementation | 3200 | 5,43 |
| *T-FER* | Iron supplementation | 1600 | 2,72 |
| *VITAFER* | Iron supplementation | 3305 | 5,61 |
| *ACFOL* | Other vitamins | 1915 | 3,25 |
| *ACIDE FOLIQUE* | Other vitamins | 100 | 0,17 |
| *ALPHAVIT* | Other vitamins | 2675 | 4,54 |
| *ALVITYL* | Other vitamins | 3838 | 6,51 |
| *CALCIUM (CAL)* | Other vitamins | 215 | 0,36 |
| *NURAVIT* | Other vitamins | 2755 | 4,68 |
| *PRONERV* | Other vitamins | 2000 | 3,39 |
| *UVESTEROL* | Other vitamins | 1600 | 2,72 |
| *VICOMBIL* | Other vitamins | 3200 | 5,43 |
