# Supplementary material for: Costs of Care of HIV-Infected Children Initiating Lopinavir/Ritonavir-Based Antiretroviral Therapy before the Age of Two in Cote d’Ivoire
Source: PLoS One. 2016 Dec 9;11(12):e0166466. doi: 10.1371/journal.pone.0166466 (PMC5147813; doi:10.1371/journal.pone.0166466)
Supplement: S2 Table — (DOCX) [file pone.0166466.s002.docx]

Supporting information

Table 2 – Breakdown of the unit costs of complementary exams in Abidjan, Côte d’Ivoire (2012)

| **Type of exam** | **Exam name** | **Cost in Ivorian francs (2012)** | **Cost in US DOLLARS (2012)** |
| --- | --- | --- | --- |
| Rapid diagnostic testing | Tuberculin skin test | 2000 | 3,39 |
|  | Malaria | 2500 | 4,24 |
|  | Other | 1000 | 1,70 |
| Blood work | Creatinine | 2500 | 4,24 |
|  | C-reactive protein | 3000 | 5,09 |
|  | Electrophoresis | 7000 | 11,88 |
|  | Blood smear | 3000 | 5,09 |
|  | Gram-negative testing | 1500 | 2,55 |
|  | Blood group determination | 2000 | 3,39 |
|  | CBC | 3000 | 5,09 |
|  | CBC + malarial RDT | 5500 | 9,33 |
|  | CBC + blood smear | 6000 | 10,18 |
|  | Protides | 4500 | 7,64 |
|  | Rhesus | 3000 | 5,09 |
|  | Unspecificied blood work | 4500 | 7,64 |
|  | Haemoglobin | 2000 | 3,39 |
|  | Transaminases | 4000 | 6,79 |
|  | Hemostatis | 4000 | 6,79 |
|  | Urea | 1500 | 2,55 |
| Laboratory / Cultures | Coproculture | 6500 | 11,03 |
|  | Lumbar puncture | 5000 | 8,49 |
|  | Parasitology | 45000 | 76,37 |
|  | BK screening | 1000 | 1,70 |
| Imaging | Electrocardiography | 125000 | 212,15 |
|  | Ultrasound | 9000 | 15,27 |
|  | Electroencephalography | 25000 | 42,43 |
|  | Endoscopy | 25000 | 42,43 |
|  | X-Ray | 8000 | 13,58 |
|  | CT-scan | 50000 | 84,86 |
|  | Cerebral CT scan | 50000 | 84,86 |
| Specialists care | Dermatologist | 5000 | 8,49 |
|  | Chiropractionner | 10000 | 16,97 |
|  | Eye specialist | 5000 | 8,49 |
|  | ENT specialist | 2000 | 3,39 |
|  | Other | 17500 | 29,70 |
| Other | Blood tranfusion | 3500 | 5,94 |
|  | Other | 2000 | 3,39 |
|  | Unkown | 4810 | 8,16 |
